# The pseudouridine synthase PUS7 is associated with stemness and represents a potential therapeutic target in triple-negative breast cancer cells

**DOI:** 10.1038/s41598-025-25684-z

**Published:** 2026-01-19

**Authors:** Lu Lu, Lian Xue, Shiyao Jiang, Guangchun He, Xiyun Deng

**Affiliations:** 1 Key Laboratory of Translational Cancer Stem Cell Research, Hunan Normal University Health Science Center, Changsha, Hunan China; 2 Department of Pathologoy, Xiangyin People’s Hospital, Yueyang, Hunan China; 3 Department of Pathology, Jiangbei Campus, The First Affiliated Hospital of Army Medical University, Chongqing, China

**Keywords:** Triple-negative breast cancer, Pseudouridine synthase, Stemness, Targeted therapy, Breast cancer, Cancer genomics

## Abstract

Triple-negative breast cancer (TNBC) represents a highly aggressive subtype of breast cancer characterized by increased recurrence rates and poor prognosis, primarily due to the lack of effective therapeutic targets. Pseudouridine synthases (PUSs) are a class of enzymes that are responsible for catalyzing the isomerization of uridine to pseudouridine in RNA, thereby contributing to cancer progression. In the present study, we examined the roles of PUSs in modulating the biological properties of TNBC using bioinformatics and experimental investigations. Increased gene expression levels of PUSs, particularly PUS7, were identified in TNBC tissues from the TCGA RNA-seq dataset and were found to be associated with unfavorable survival of TNBC patients. In addition, increased protein levels of PUS7 were identified in TNBC patient tissues and cell lines compared with non-TNBC. The increased PUS7 expression was in line with the stemness of TNBC cells. Knockdown of PUS7 in MDA-MB-231 and MDA-MB-468 cells inhibited stemness, migration, and colony formation. Transfection with a PUS7-Mut construct, which eliminated the enzymatic activity of PUS7, reversed the stimulating effects of PUS7 on stemness, migration, and colony formation in TNBC cells. This study highlights the influence of PUS7 on the biological properties of TNBC through its enzymatic activity, providing valuable insights and potential avenues for the identification of effective therapeutic targets for TNBC.

## Introduction

Breast cancer is the second most prevalent malignancy and has become the primary cause of cancer-related mortality among women globally^1^. Among various breast cancer subtypes, triple-negative breast cancer (TNBC) is notable for the absence of estrogen receptor (ER), progesterone receptor (PR), and human epidermal growth factor receptor 2 (HER2). TNBC is characterized by high recurrence rates and unfavorable prognosis due to the lack of effective treatment targets^2^. Deeper understanding of the biology behind TNBC has led to the development of innovative targeted therapies, such as PARP inhibitors, antibody–drug conjugates, and immune checkpoint inhibitors, offering new treatment avenues for TNBC patients^3,4^. While these therapies have shown promise in a portion of TNBC patients, the majority still do not benefit from these novel therapies. For example, in the KEYNOTE-355 clinical study, the anti-PD1 antibody pembrolizumab combined with chemotherapy has been shown to extend the progression-free survival of metastatic TNBC patients with a combined positive score of ≥ 10 by an average of only 4.1 months^5^. Thus, the identification of novel and effective therapeutic targets remains a crucial focus in TNBC research, offering significant implications for improving patient outcomes.

Chemical modifications in RNA impact the transcription process by altering charge, base pairing, secondary structure, and protein-RNA interactions. Such modifications ultimately dictate gene expression by regulating RNA processing, localization, translation, and decay^6^. Due to the ability of RNA modifications to modulate various aspects of RNA metabolism and influence protein synthesis rates in cancer, RNA modifications have emerged as a crucial regulatory mechanism and a novel target for cancer therapy^7,8^. Pseudouridine (Ψ) modification, the most abundant modification found in RNA, involves the action of pseudouridine synthases (PUSs)^6,9^, a group of enzymes that catalyze the pseudouridylation of substrate RNA molecules^10^. Recent studies have established a definite link between RNA pseudouridylation, PUS enzymes, and the progression of various types of cancers^11,12^. Specifically, PUS7 contributes to tumorigenesis by modifying RNA at designated sites, catalyzing Ψ modification across different types of RNA molecules^13,14^.

Among the various types of PUSs, PUS7 stands out for its ability to regulate translation of stem cells through modifications at various sites on mRNAs^14^. PUS7 has been shown to promote tumor initiation and progression in diverse cancer types including glioblastoma^15^ and colorectal cancer^16^. Additionally, PUS7 has been identified as a potential biomarker for certain cancer types such as ovarian cancer^17^. However, the diverse expression patterns and prognostic significance of PUSs in cancer are beginning to be appreciated. Additional research is needed to determine the regulatory mechanisms and biological functions of PUS7 in solid tumors, particularly in TNBC. In the present study, we aimed to investigate the impact of PUS7 on the biological properties of TNBC cells with a focus on stemness-related processes. We found the expression levels of PUS7 were increased in TNBC, which were associated with poor prognosis in TNBC patients. We also show a link between PUS7 and TNBC stemness depending on the enzymatic activity of PUS7. Our findings provide new insights and directions for the development of pseudouridine synthase-based targeted therapeutic strategies for TNBC.

## Materials and methods

### TCGA-BRCA RNA-seq and clinical data

The TCGA RNA-seq and clinical data of breast cancer patients were obtained from the Genomic Data Commons (GDC) Data Portal (https://portal.gdc.cancer.gov/). This study analyzed a total of 1,102 cases of breast cancer samples (231 TNBC and 871 non-TNBC cases) from the TCGA-BRCA database. The PAM50 subtyping classifier was utilized to distinguish between basal-like (a surrogate for TNBC) and other breast cancer subtypes.

### GEPIA2 analysis

The relationship of PUS7 with ribosome biogenesis-related and stemness-related gene sets in breast cancer and TNBC patients were analyzed using the online database Gene Expression Profiling Interactive Analysis 2 (GEPIA2) (http://gepia2.cancer-pku.cn/).

### Kaplan–Meier plotter

Prognostic values of PUSs in breast cancer and in TNBC patients was analyzed using the Kaplan–Meier Plotter (http://kmplot.com/analysis/).

### Functional enrichment analysis

A ribosome biogenesis-related gene set containing 548 genes was downloaded from the Molecular Signatures Database (MSigDB) (https://www.gsea-msigdb.org/gsea/msigdb/). Gene Ontology (GO) analysis, Venn diagram analysis, and Gene Set Enrichment Analysis (GSEA) were performed using the R/Bioconductor package “*clusterProfiler*”. For enriched gene sets, a normalized *P* < 0.05 and a false discovery rate (FDR) q < 0.25 were considered statistically significant. The protein–protein interaction (PPI) network was generated using the STRING (https://string-db.org/) database. The relationship between PUS7 expression and stemness-related genes in breast cancer and TNBC patients was analyzed through the TIMER (https://cistrome.shinyapps.io/timer/) website.

### Cell culture

TNBC cell lines (MDA-MB-231, MDA-MB-468, BT549, and HCC1806) and non-TNBC cell lines (ZR-75-1, MDA-MB-453, T47D, and BT474) were obtained from the CAS Shanghai Cell Resources Center and cultured following the provider’s guidelines. None of these cell lines are included in the ICLAC database of commonly cross-contaminated or misidentified cell lines (http://iclac.org/databases/cross-contaminations/).

### Sphere-forming assay

The cells were maintained in ultra-low attachment 6-well plates (2500–5000 cells/well) in serum-free stem cell medium to obtain sphere-forming cells (SFCs). The culture medium consisted of DMEM/F12 (Life Technologies, Grand Island, NY, USA) supplemented with 1 × B27 (Life Technologies), 20 ng/mL EGF (Prospec, East Brunswick, NJ, USA), 20 ng/mL bFGF (Prospec), 0.4% BSA (Sigma-Aldrich, St Louis, MO, USA), and 4 µg/mL Insulin (Genview, Pompano Beach, FL, USA). The sphere-forming capacity of breast cancer cells was measured by plating the parental cells in serum-free stem cell culture medium and calculating the areas of tumorspheres formed 5 days after treatment as described in our recent publication^18^.

### Cell transfection

PUS7 siRNA was synthesized by Biological Engineering Shanghai Co., Ltd., with sequence details available in Supplementary Table S1. The cells were seeded in a 6-well plate, grown to around 80% confluency, and subjected to transfection for transient PUS7 knockdown using the DharmaFECT transfection reagent as instructed by the manufacturer. Wild-type PUS7 (PUS7-WT) and PUS7 D294A mutant (PUS7-Mut)^16^ plasmids were synthesized by Hunan Jima Co., Ltd., with details also available in Supplementary Table S1. The D294A mutation abolishes enzymatic activity of PUS7 but does not affect its protein stability or intracellular localization^16^. These plasmids were transfected into exponentially growing cells using Lipofectamine™ 3000 (Thermo Fisher Scientific, Waltham, MA, USA). 48 h after transfection, puromycin was added at a final concentration of 1 µg/mL for selection or 0.5 µg/mL for maintenance of the culture. PUS7 silencing or overexpression efficiency was confirmed via western blot analysis.

### Immunohistochemistry

For immunohistochemistry (IHC), the PV-9000 plus poly-HRP anti-mouse/rabbit IgG detection system was used according to our previous study. The details of primary antibodies for IHC are summarized in Supplementary Table S2. Tissue sections were quantified using the automated quantitative pathology imaging system (Vectra, PerkinElmer, Hopkinton, MA, USA) and the immunoreactivity score was calculated accordingly. This module determined staining intensity (average grayscale value) and staining area (percentage of positive area) as measurement indices, resulting in four scoring categories: strong positive (3 point), positive (2 point), weak positive (1 point), and negative (0 point).

### Western blot analysis

Whole cell lysates were prepared and quantified according to the standard procedure. Western blot analysis was performed by separating the cell lysates on 10% or 12% SDS-PAGE gels, followed by electroblotting, primary and secondary antibody incubation, and ECL development. When necessary, band intensities were quantified using the Bio-Rad Image Lab software and normalized to the house-keeping protein. The information of primary antibodies for western blot analysis is also available in Supplementary Table S2.

### Transwell migration assay

Cell migration assay was conducted with PET membranes (8 μm pore size, 24-well plate) without Matrigel coating (Corning, Tewksbury, MA, USA) for migration. Single cell suspension in serum-free medium was added to the upper wells (20,000 cells/well), while migration-inducing medium (with 10% FBS) was added to the lower wells. After 24 h, the cells on the top surface of the chambers were scraped off with a cotton swab and the cells on the lower surface of the membranes were fixed with methanol and stained with 0.1% crystal violet solution. The cells that penetrated the membrane were evaluated microscopically and quantified from three random fields per membrane.

### MTT assay

The cells were transfected with non-targeting siRNA (siNC) or PUS7-targeting siRNAs (siPUS7-1, siPUS7-2) in 6-well plates at a density of 5 × 10^4^ cells/well. After 24 h of transfection, 20 μL of MTT solution (5 mg/mL in PBS) was added to each well, and the plates were incubated at 37 °C for 4 h. Absorbance at 490 nm was measured using a microplate reader, with cell viability normalized to the siNC control group.

### Colony formation assay

For the colony formation assay, the cells in single-cell suspension were seeded in 6-well plates (1000 cells/plate), with the medium refreshed every 3 days. After 14 days of incubation, the medium was discarded and the colonies were washed with PBS, and then stained with 0.1% crystal violet solution for 15 min. The colonies were then photographed, followed by counting and quantification.

### Scratch wound healing assay

After trypsin digestion, the cells were dispersed into a single state and then seeded into a 6-well plate and cultured in a regular oxygenated, 5% CO_2_, 37 °C incubator. Once the cells covered around 80% of the bottom of the 6-well plate, they were rinsed twice with PBS, and then scratched using a 200-μL pipettor tip. Subsequently, the cells were rinsed twice with PBS to remove the scratched cells, and 2 mL of serum-free medium was added. Images were taken at 0, 12, and 24 h, and the average distance between scratches was calculated to analyze the cell migration rate.

### RNA dot blot assay

Total RNA was prepared using the TRIzol reagent according to the manufacturer’s procedure and diluted in RNase-free water to a working concentration of 400 ng/μL. After denaturation at 95 °C for 10–15 min, the RNA samples were cooled on ice to prevent the formation of secondary structures. The nylon membrane with a grid lightly marked on it was cut and transferred to a hybridization box lined with sealing film. The RNA samples were mixed, dispensed onto the membrane, and allowed to air dry for 5 min or longer. The membrane was exposed to UV light for 10 min, washed in TBST, and blocked for 90 min. The membrane was incubated with an anti-pseudouridine antibody, rinsed, incubated with the HRP-conjugated secondary antibody, washed, and developed using the ECL system, followed by staining with methylene blue for normalization.

### Statistical analysis

The quantitative data were expressed as mean ± SEM. For pairwise comparisons, two-tailed unpaired Student’s *t*-test was used; one-way analysis of variance (ANOVA) followed by Tukey’s post-hoc test was used for multiple comparison correction. *P* values < 0.05 were considered statistically significant.

## Results

### Expression of PUSs in breast cancer patient tissues and their association with patient survival

**Fig. 1:**
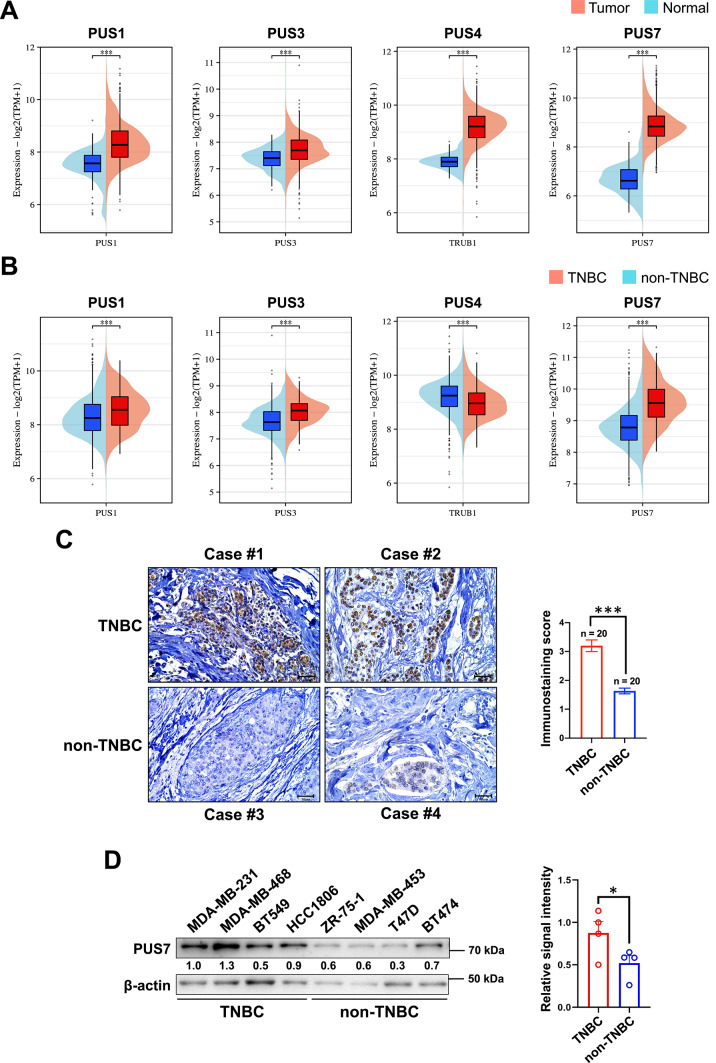
Expression levels of PUSs in breast cancer and TNBC patient tissues and cells. (**A**, **B**) The expression levels of PUS1, PUS3, PUS4, and PUS7 analyzed between breast cancer and normal breast tissues (**A**) and between TNBC and non-TNBC tissues (**B**) from the TCGA-BRCA dataset. (**C**) Immunohistochemistry performed to examine the protein level of PUS7 in paraffin-embedded tissues from TNBC and non-TNBC patients (n = 20/group). Representative images and statistical graphs were illustrated. (**D**) The protein level of PUS7 in TNBC and non-TNBC cell lines detected by western blotting. *P* values were denoted as * for *P* < 0.05 and *** for *P* < 0.001.

To investigate the expression levels of PUSs in breast cancer and TNBC patients and their association with patient survival, we downloaded the TCGA-BRCA dataset from the GDC Data Portal, followed by analysis with the ggplot2 package. The mRNA levels of key PUS family members, i.e., PUS1, PUS3, PUS4, and PUS7, were significantly higher in breast cancer patient tissues compared with normal breast tissues (Fig. 1A). Furthermore, compared with non-TNBC, PUS1, PUS3, and PUS7 mRNAs exhibited higher expression, while PUS4 showed lower expression in TNBC tissues (Fig. 1B). Higher expression of the PUS7 gene in TNBC compared with non-TNBC tissues was confirmed using the GEPIA2 web-based analysis (Supplementary Fig. 1).

Based on the above finding that the PUS7 gene was expressed at a much higher level in TNBC compared with non-TNBC tissues, we performed IHC on paraffin-embedded tissue sections to investigate the protein level of PUS7 in breast cancer patient tissues. In agreement with the mRNA expression data, higher protein level of PUS7 was revealed in TNBC compared with non-TNBC tissues (Fig. 1C). We further investigated the protein level of PUS7 in a panel of breast cancer cell lines. As expected, the PUS7 protein was observed at a higher level in TNBC compared with non-TNBC cell lines (Fig. 1D).

**Fig. 2:**
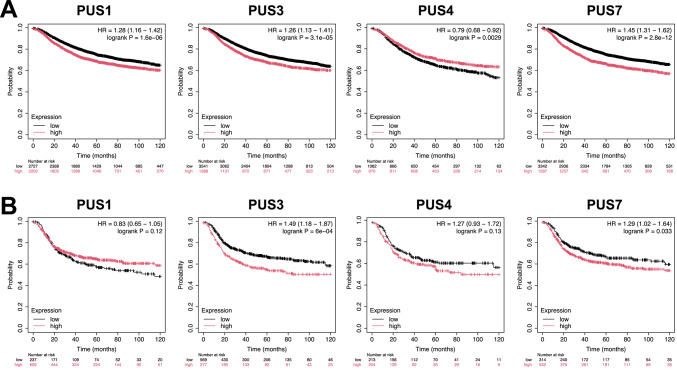
Association of the expression levels of PUSs with patient survival in breast cancer and TNBC. (**A**, **B**) Association between the expression levels of PUS1, PUS3, PUS4 and PUS7 and the prognosis in breast cancer and TNBC patients. The prognostic values of PUS1, PUS3, PUS4, and PUS7 were analyzed in breast cancer (**A**) and TNBC (**B**) patients using Kaplan–Meier Plotter.

Next, we analyzed the correlation between the expression of PUS genes in breast cancer and TNBC patients and their association with patient survival through Kaplan–Meier Plotter. While higher expression of PUS4 was associated with more favorable prognosis, higher expression of PUS1, PUS3, and PUS7 in breast cancer patients was correlated with poorer prognosis (Fig. 2A). In TNBC patients, higher expression of PUS3 and PUS7 was associated with poorer prognosis, although the expression of PUS1 and PUS4 was not significantly correlated with patient survival (Fig. 2B). These results suggest that higher expression of PUS7, and possibly PUS3, predicts poorer prognosis in patients with breast cancer and TNBC. Given the more significant expression of PUS7 in TNBC tissues and cell lines and the association of PUS7 expression with unfavorable prognosis of TNBC patients, PUS7 was selected for further investigation.

### PUS7 is correlated with ribosome biogenesis and the stemness in TNBC

**Fig. 3:**
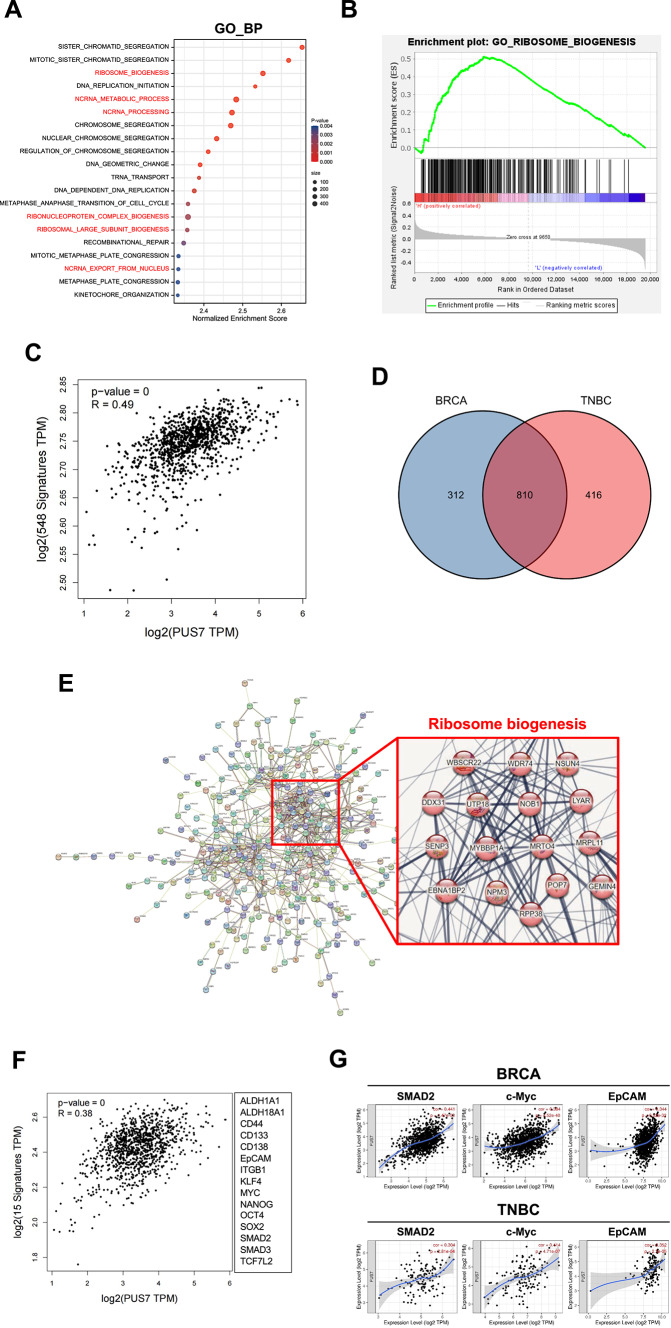
Correlation of gene expression between PUS7 and the genes associated with ribosome biogenesis and the stemness in breast cancer and TNBC patients. (**A**) GO_BP pathway enrichment of TNBC patients in the TNBC-BRCA dataset with high PUS7 expression. (**B**) GSEA analysis of the correlation between PUS7 expression and the ribosome biogenesis pathway in TNBC patients. (**C**) The correlation of gene expression between PUS7 and a gene set consisting of 548 genes related to ribosome biogenesis in breast cancer patients analyzed using the GEPIA2 website. (**D**) The Venn diagram showing the relationship between the genes highly expressed in breast cancer and TNBC patients. (**E**) A PPI network diagram of the 416 genes highly expressed in TNBC patients generated by STRING analysis. (**F**) The correlation of gene expression between PUS7 and its relevance to the stemness-related gene set in breast cancer patients using the GEPIA2 website. The 15-gene signature associated with stemness was presented on the right. (**G**) The correlation of gene expression between PUS7 and stemness genes in breast cancer and TNBC patients evaluated using the TIMER website.

In order to reveal the correlation between PUS7 expression and intracellular signaling, the 231 TNBC patients contained in the TCGA-BRCA dataset were divided into high- and low-expression groups based on the median expression level of PUS7. The differentially expressed genes were then obtained between the high- and low-expression groups. GO_BP analysis revealed that the differentially expressed genes were mainly enriched in ribosome biogenesis-related pathways including ribosome biogenesis, ncRNA metabolic process, ncRNC processing, etc. (Fig. 3A). The correlation between PUS7 expression and the ribosome biogenesis pathway in TNBC was confirmed by the GSEA enrichment plot (Fig. 3B). We further analyzed the correlation of PUS7 with the ribosome biogenesis-related gene set^18^ in breast cancer patients using the GEPIA2 website and a correlation coeffient (R) of 0.49 was obtained (*P* < 0.05) (Fig. 3C). These results suggest a definite link between PUS7 and the ribosome biogenesis pathway in breast cancer.

Next, we investigated the relationships between PUS7 expression and differentially expressed genes in breast cancer and TNBC. While 312 genes were correlated solely with breast cancer and 810 genes were common to both breast cancer and TNBC, 416 genes were upregulated exclusively in TNBC patients (Fig. 3D). Further PPI network analysis revealed significant enrichment of these 416 genes in the ribosome biogenesis pathway (Fig. 3E). Because of the recently identified close correlation of ribosome biogenesis and the stemness in TNBC^18^, we further analyzed the correlation of PUS7 with the stemness-related gene set in breast cancer patients using the GEPIA2 website. A correlation coefficient (R) of 0.38 was revealed between PUS7 expression and the stemness-related gene set in breast cancer patients (*P* < 0.05) (Fig. 3F). High-level correlation between PUS7 and three key stemness-related genes (SMAD2, c-Myc, EpCAM) was further revealed in breast cancer and TNBC patients through TIMER analysis (Fig. 3G). These results suggest that PUS7 is highly correlated with the stemness feature in TNBC.

### Knocking down PUS7 inhibits the stemness properties of TNBC cells

**Fig. 4:**
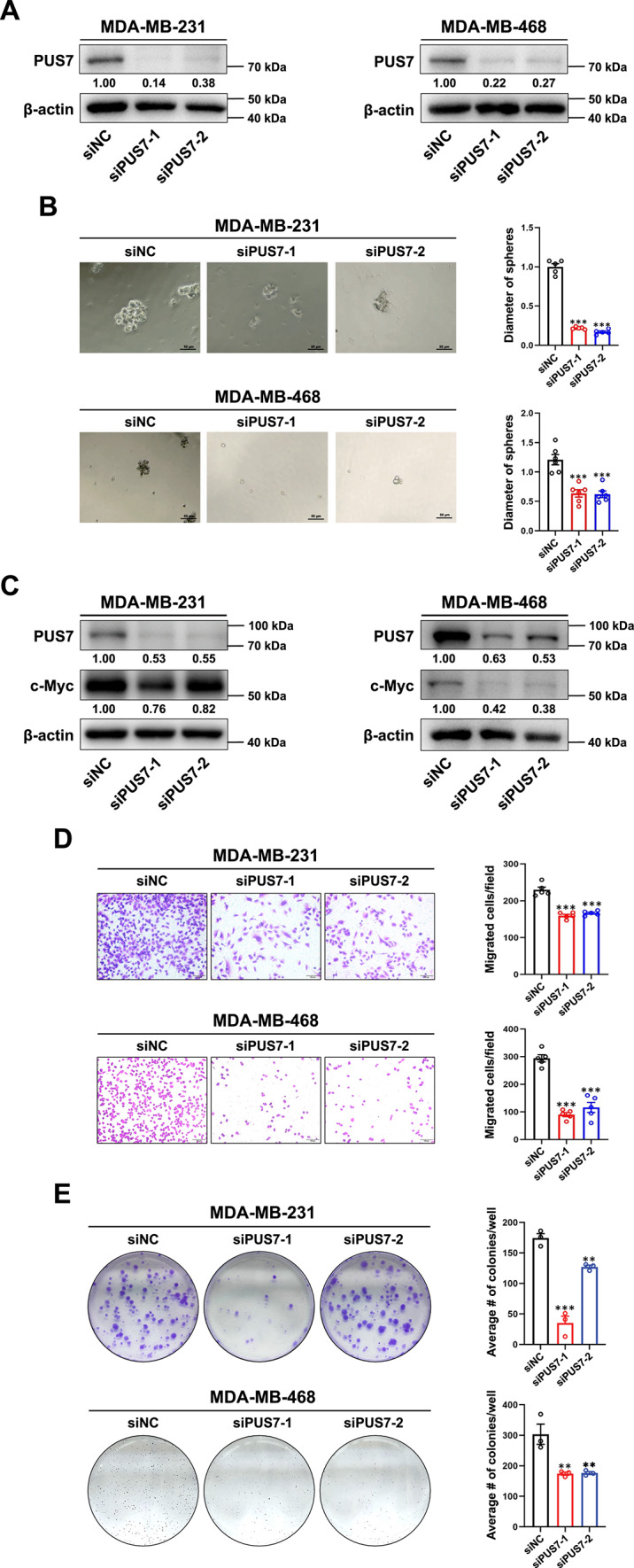
PUS7 knockdown inhibits migration and proliferative ability in TNBC cells. (**A**) The knockdown efficiency of PUS7 in MDA-MB-231 and MDA-MB-468 cells examined by western blotting. (**B**) The impact of PUS7 on the stemness of MDA-MB-231 and MDA-MB-468 cells assessed by sphere formation assay. (**C**) The effect of PUS7 on c-Myc, a stemness marker, in MDA-MB-231 and MDA-MB-468 cells investigated by western blotting. (**D**) The effect of PUS7 on the migratory ability of MDA-MB-231 and MDA-MB-468 cells examined via transwell experiments. (**E**) The proliferative capacity of MDA-MB-231 and MDA-MB-468 cells upon PUS7 knockdown analyzed through colony formation. Scale bars = 100 μm. *P* values were denoted as ** for *P* < 0.01 and *** for *P* < 0.001.

To further investigate the impact of PUS7 on TNBC stemness, PUS7 was knocked down in MDA-MB-231 and MDA-MB-468 cell lines (Fig. 4A). After culturing in stem cell medium for 7 days, tumorsphere diameters were measured as an indication of the impact of PUS7 knockdown on the stemness of TNBC cells. We found a significant inhibitory effect on tumorsphere formation in both MDA-MB-231 and MDA-MB-468 TNBC cell lines after PUS7 knockdown (Fig. 4B). Additionally, western blotting in both MDA-MB-231 and MDA-MB-468 TNBC cell lines revealed a relative decrease in c-Myc expression after PUS7 knockdown (Fig. 4C). Next, we assessed the effects of PUS7 knockdown on the migratory ability of TNBC cells using transwell and scratch wound healing assays. There was significant reduction in cell migratory ability upon PUS7 knockdown in MDA-MB-231 and MDA-MB-468 cells (Fig. 4D, Supplementary Fig. 2). We quantified relative cell viability (normalized to siNC) using an MTT assay, which showed no significant differences between the PUS7-knockdown group and the siNC control in either MDA-MB-231 or MDA-MB-468 cells (Supplementary Fig. 3). These results validate that the reduced migration observed in PUS7-knockdown cells was attributed to impaired migratory ability rather than changes in proliferation rate. We further revealed that knocking down PUS7 had an inhibitory effect on the proliferation of MDA-MB-231 and MDA-MB-468 cells, as demonstrated by decreased ability to form cell colonies in PUS7 knockdown cells (Fig. 4E). Together, these results imply a role for PUS7 in TNBC stemness possibly through c-Myc regulation.

### Promotion of TNBC stemness by PUS7 depends on its enzymatic activity

Lastly, we determined whether the enzymatic activity of PUS7 is required for its promoting effects on TNBC stemness. It has been demonstrated that D294 of PUS7 is a key site in determining the enzymatic activity of PUS7^16^. Consequently, a wild-type PUS7 overexpression plasmid (PUS7-WT) and a D294A mutant plasmid (PUS7-Mut) were constructed based on the PUS7 sequence. PUS7-WT, PUS7-Mut, and the vector control plasmids were transfected into MDA-MB-231 and MDA-MB-468 cell lines. Western blotting confirmed high expression of PUS7 at the protein level in the cells transfected with the PUS7-WT or PUS7-Mut plasmid (Supplementary Fig. 4). Furthermore, RNA dot blot analysis demonstrated a significant decrease in the level of Ψ in PUS7-Mut cells compared with the PUS7-WT group, suggesting successful inhibition of the enzymatic activity of PUS7 through site-directed mutagenesis (Supplementary Fig. 5).

**Fig. 5:**
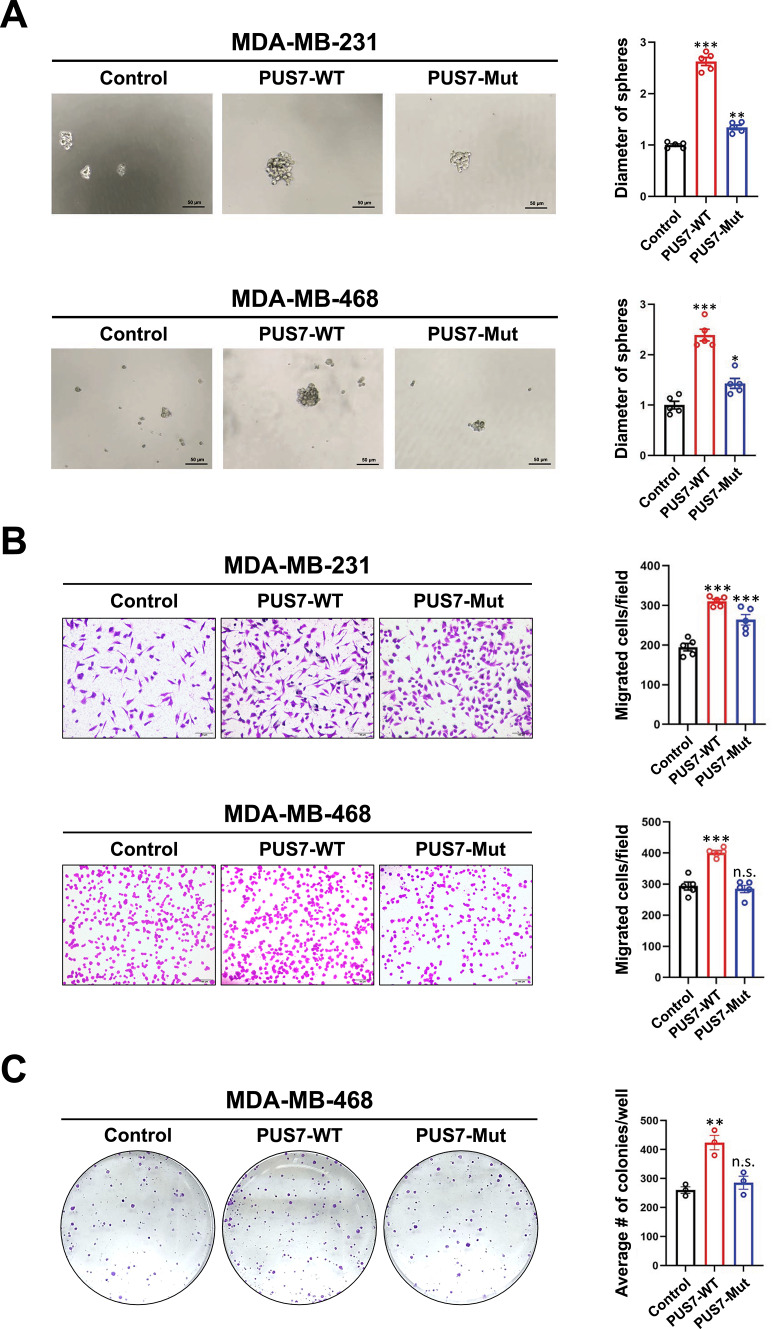
The effects PUS7 D294A mutation on the malignant phenotypes of TNBC cells. (**A**) The effect of PUS7 D294A mutation on the tumorsphere formation ability of MDA-MB-231 and MDA-MB-468 cells assessed via the tumorsphere formation assay. (**B**) The effect of PUS7 D294A mutation on the migratory ability of MDA-MB-231 and MDA-MB-468 cells examined by the transwell migration assay. (**C**) The effect of PUS7 D294A mutation on the proliferation ability of MDA-MB-468 cells analyzed using the colony formation assay. Scale bars = 100 μm. *P* values were denoted as * for *P* < 0.05, ** for *P* < 0.01, *** for *P* < 0.001, and n.s. for not significant.

We then asked whether inactivating PUS7 enzymatic activity would affect the stemness-related properties of TNBC cells. After transfection with the PUS7-WT, PUS7-Mut, or the vector control plasmid, the effects of PUS7-WT and PUS7-Mut on TNBC cell stemness were analyzed by tumorsphere formation assay. We found that transfection with PUS7-Mut significantly reversed the promoting effect of PUS7-WT on tumorsphere formation in TNBC cells (Fig. 5A). Similary, transwell migration assay revealed that the PUS7-Mut reversed the stimulatory effect of PUS7-WT on the migratory ability of TNBC cells (Fig. 5B). In addition, while the proliferation of MDA-MB-468 cells transfected with PUS7-WT was increased compared with the control cells, this increase in the proliferative ability was reversed in the cells transfected with PUS7-Mut (Fig. 5C). These results suggest that the enzymatic activity of PUS7 is a main factor for its activity in promoting malignant phenotypes including stemness in TNBC cells.

## Discussion

TNBC, as the most aggressive subtype of breast cancer, exhibits poor response to traditional therapy due to the absence of hormone receptors and HER2^19^. Targeted therapeutic strategies taking into account the heterogeneity of the tumor microenvironment have significantly improved the objective response rates of advanced TNBC patients resistant to multiple therapies^20–26^. Nonetheless, these novel strategies have benefited only a subset of TNBC patients and treatment outcomes of the majority of these patients remain unsatisfactory, emphasizing the need to identify more robust treatment targets for TNBC.

RNA modifications can enhance genetic information diversity and influence protein synthesis efficiency and fidelity^27,28^. PUS7 is recognized as an important pseudouridine synthase involved in cancer biology among other pseudouridine synthases^29^. In this study, we investigated the impact of PUS7 on TNBC stemness and malignant phenotypes. Our study reveals a role for PUS7 as a pivotal enzyme responsible for pseudouridine modification in TNBC cells. Limited research currently exists on the connection between PUS7 and solid tumors except for glioblastoma and colorectal cancer. In glioblastoma, PUS7 targets the tRNA for pseudouridylation, affecting the interferon-STAT1 pathway and cell growth by reducing Tyk2 translation^15^. In colorectal cancer, PUS7 facilitates cancer cell migration through the HSP90/PUS7/LASP1 pathway, enhances proliferation via SIRT1 activating the Wnt/β-catenin pathway, and stimulates proliferation and invasion through the PI3K/AKT/mTOR signaling pathway^16,30,31^.

Elucidating the molecular mechanisms involved in the modulation of malignant phenotypes including stemness by PUS7 may reveal potential therapeutic targets, providing a foundation for the development of novel treatment strategies for TNBC. Given the conserved catalytic domain of the PUS family, rational drug design guided by structural studies could enable the development of inhibitors targeting its enzymatic activity. Alternatively, nucleic acid–based approaches, including RNA interference, antisense oligonucleotides, or CRISPR/Cas9-mediated gene editing, may serve as preclinical tools to explore the effects of PUS7 inhibition. In addition, combination strategies with existing TNBC-targeted therapies should be considered. PARP inhibitors (e.g., olaparib, talazoparib) are effective in BRCA-mutant TNBC^32^, but resistance frequently develops. Thus, combining PUS7-targeted approaches with PARP inhibition could expand therapeutic benefit beyond BRCA-mutant tumors and potentially overcome resistance. Furthermore, the synergistic effects of PUS7 suppression with other agents such as those targeting DNA repair, cell cycle, or immune checkpoints should also be investigated. Future investigations should aim to integrate transcriptomic and proteomic analyses to assess the broader impact of PUS7 on cell signaling within the tumor microenvironment, offering insights into its role in tumor heterogeneity and resistance to therapy. The ultimate goal is to decipher the specific roles and molecular mechanisms of PUS7 in malignant phenotypes including stemness in TNBC, rendering PUS7 a viable target for TNBC therapy.

Our findings indicate a crucial relationship between the enzymatic activity of PUS7 and its role in promoting TNBC cell growth. By reversing this effect through targeted mutations, the study opens new avenues for potential therapeutic strategies aimed at TNBC. These insights not only underscore the importance of PUS7 in cancer biology but also pave the way for future investigations into the molecular mechanisms underpinning TNBC progression and the development of more effective, enzyme-targeted interventions. These results suggest that PUS7 is important in determining the stemness properties of TNBC cells and can be used as an innovative target for precision therapy of TNBC patients with high a level of PUS7 expression.

## Data Availability

The datasets generated during this study are not publicly available due to the institutional restrictions but are available from the corresponding author on reasonable request.

## Abbreviations

BRCA: Breast cancer
ER: Estrogen receptor
GEPIA2: Gene expression profiling interactive analysis 2
GSEA: Gene set enrichment analysis
HER2: Human epidermal growth factor 2
IHC: Immunohistochemistry
PR: Progesterone receptor
PUS: Pseudouridine synthase
RFS: Relapse-free survival
TCGA: The Cancer Genome Atlas
TIMER: Tumor immune estimation resource
TNBC: Triple-negative breast cancer
Ψ: Pseudouridine

## References

[1] Bray, F. et al. Global cancer statistics 2022: GLOBOCAN estimates of incidence and mortality worldwide for 36 cancers in 185 countries. *CA Cancer J. Clin.* **74**, 229–263. 10.3322/caac.21834 (2024). 38572751 10.3322/caac.21834

[2] Yin, L., Duan, J. J., Bian, X. W. & Yu, S. C. Triple-negative breast cancer molecular subtyping and treatment progress. *Breast Cancer Res.* **22**, 61. 10.1186/s13058-020-01296-5 (2020). 32517735 10.1186/s13058-020-01296-5 PMC7285581

[3] Bianchini, G., De Angelis, C., Licata, L. & Gianni, L. Treatment landscape of triple-negative breast cancer—expanded options, evolving needs. *Nat. Rev. Clin. Oncol.* **19**, 91–113. 10.1038/s41571-021-00565-2 (2022). 34754128 10.1038/s41571-021-00565-2

[4] Keenan, T. E. & Tolaney, S. M. Role of immunotherapy in triple-negative breast cancer. *J. Natl. Compr. Canc. Netw.* **18**, 479–489. 10.6004/jnccn.2020.7554 (2020). 32259782 10.6004/jnccn.2020.7554

[5] Cortes, J. et al. Pembrolizumab plus chemotherapy versus placebo plus chemotherapy for previously untreated locally recurrent inoperable or metastatic triple-negative breast cancer (KEYNOTE-355): A randomised, placebo-controlled, double-blind, phase 3 clinical trial. *Lancet* **396**, 1817–1828. 10.1016/S0140-6736(20)32531-9 (2020). 33278935 10.1016/S0140-6736(20)32531-9

[6] Roundtree, I. A., Evans, M. E., Pan, T. & He, C. Dynamic RNA modifications in gene expression regulation. *Cell* **169**, 1187–1200. 10.1016/j.cell.2017.05.045 (2017). 28622506 10.1016/j.cell.2017.05.045 PMC5657247

[7] Rapino, F., Delaunay, S., Zhou, Z., Chariot, A. & Close, P. tRNA Modification: Is cancer having a wobble?. *Trends Cancer* **3**, 249–252. 10.1016/j.trecan.2017.02.004 (2017). 28718436 10.1016/j.trecan.2017.02.004

[8] Dai, D., Wang, H., Zhu, L., Jin, H. & Wang, X. N6-methyladenosine links RNA metabolism to cancer progression. *Cell Death Dis.* **9**, 124. 10.1038/s41419-017-0129-x (2018). 29374143 10.1038/s41419-017-0129-x PMC5833385

[9] Carlile, T. M. et al. Pseudouridine profiling reveals regulated mRNA pseudouridylation in yeast and human cells. *Nature* **515**, 143–146. 10.1038/nature13802 (2014). 25192136 10.1038/nature13802 PMC4224642

[10] Rintala-Dempsey, A. C. & Kothe, U. Eukaryotic stand-alone pseudouridine synthases—RNA modifying enzymes and emerging regulators of gene expression?. *RNA Biol* **14**, 1185–1196. 10.1080/15476286.2016.1276150 (2017). 28045575 10.1080/15476286.2016.1276150 PMC5699540

[11] Borchardt, E. K., Martinez, N. M. & Gilbert, W. V. Regulation and function of RNA pseudouridylation in human cells. *Annu. Rev. Genet.* **54**, 309–336. 10.1146/annurev-genet-112618-043830 (2020). 32870730 10.1146/annurev-genet-112618-043830 PMC8007080

[12] Karijolich, J., Yi, C. & Yu, Y. T. Transcriptome-wide dynamics of RNA pseudouridylation. *Nat. Rev. Mol. Cell Biol.* **16**, 581–585. 10.1038/nrm4040 (2015). 26285676 10.1038/nrm4040 PMC5694666

[13] Zhang, D. Y., Ming, G. L. & Song, H. PUS7: A targetable epitranscriptomic regulator of glioblastoma growth. *Trends Pharmacol. Sci.* **42**, 976–978. 10.1016/j.tips.2021.10.002 (2021). 34657723 10.1016/j.tips.2021.10.002

[14] Gilbert, W. V., Bell, T. A. & Schaening, C. Messenger RNA modifications: Form, distribution, and function. *Science* **352**, 1408–1412. 10.1126/science.aad8711 (2016). 27313037 10.1126/science.aad8711 PMC5094196

[15] Cui, Q. et al. Targeting PUS7 suppresses tRNA pseudouridylation and glioblastoma tumorigenesis. *Nat. Cancer* **2**, 932–949. 10.1038/s43018-021-00238-0 (2021). 35121864 10.1038/s43018-021-00238-0 PMC8809511

[16] Song, D. et al. HSP90-dependent PUS7 overexpression facilitates the metastasis of colorectal cancer cells by regulating LASP1 abundance. *J. Exp. Clin. Cancer Res.* **40**, 170. 10.1186/s13046-021-01951-5 (2021). 33990203 10.1186/s13046-021-01951-5 PMC8120699

[17] Li, H. et al. The identification of RNA modification gene PUS7 as a potential biomarker of ovarian cancer. *Biology* **10**, 1130. 10.3390/biology10111130 (2021). 34827123 10.3390/biology10111130 PMC8615213

[18] Zheng, C. et al. Dysregulated ribosome biogenesis is a targetable vulnerability in triple-negative breast cancer: MRPS27 as a key mediator of the stemness-inhibitory effect of lovastatin. *Int. J. Biol. Sci.* **20**, 2130–2148. 10.7150/ijbs.94058 (2024). 38617541 10.7150/ijbs.94058 PMC11008279

[19] Ferrari, P. et al. Molecular mechanisms, biomarkers and emerging therapies for chemotherapy resistant TNBC. *Int. J. Mol. Sci.* **23**, 1665. 10.3390/ijms23031665 (2022). 35163586 10.3390/ijms23031665 PMC8836182

[20] Jiang, Y. Z. et al. Molecular subtyping and genomic profiling expand precision medicine in refractory metastatic triple-negative breast cancer: The FUTURE trial. *Cell Res* **31**, 178–186. 10.1038/s41422-020-0375-9 (2021). 32719455 10.1038/s41422-020-0375-9 PMC8027015

[21] Jiang, Y. Z. et al. Genomic and transcriptomic landscape of triple-negative breast cancers: Subtypes and treatment strategies. *Cancer Cell* **35**, 428–440. 10.1016/j.ccell.2019.02.001 (2019). 30853353 10.1016/j.ccell.2019.02.001

[22] Liu, Y. et al. Subtyping-based platform guides precision medicine for heavily pretreated metastatic triple-negative breast cancer: The FUTURE phase II umbrella clinical trial. *Cell Res.* **33**, 389–402. 10.1038/s41422-023-00795-2 (2023). 36973538 10.1038/s41422-023-00795-2 PMC10156707

[23] Yang, F. et al. Ferroptosis heterogeneity in triple-negative breast cancer reveals an innovative immunotherapy combination strategy. *Cell Metab.* **35**, 84–100. 10.1016/j.cmet.2022.09.021 (2023). 36257316 10.1016/j.cmet.2022.09.021

[24] Fan, L. et al. Optimising first-line subtyping-based therapy in triple-negative breast cancer (FUTURE-SUPER): A multi-cohort, randomised, phase 2 trial. *Lancet Oncol.* **25**, 184–197. 10.1016/S1470-2045(23)00579-X (2024). 38211606 10.1016/S1470-2045(23)00579-X

[25] Rao, X. et al. Integrating radiosensitivity index and triple-negative breast cancer subtypes reveals SERPINB5 as a radioresistance biomarker in triple-negative breast cancer. *Clin. Transl. Med.* **14**, e1787. 10.1002/ctm2.1787 (2024). 39113221 10.1002/ctm2.1787 PMC11306282

[26] Yang, Y. et al. Dual inhibition of CDK4/6 and CDK7 suppresses triple-negative breast cancer progression via epigenetic modulation of SREBP1-regulated cholesterol metabolism. *Adv. Sci.*10.1002/advs.202413103 (2024). 10.1002/advs.202413103 PMC11791979 39656925

[27] Davalos, V., Blanco, S. & Esteller, M. SnapShot: Messenger RNA modifications. *Cell* **174**, 498–498. 10.1016/j.cell.2018.06.046 (2018). 30007421 10.1016/j.cell.2018.06.046

[28] Jonkhout, N. et al. The RNA modification landscape in human disease. *RNA* **23**, 1754–1769. 10.1261/rna.063503.117 (2017). 28855326 10.1261/rna.063503.117 PMC5688997

[29] Rasmuson, T. & Bjork, G. R. Urinary excretion of pseudouridine and prognosis of patients with malignant lymphoma. *Acta Oncol.* **34**, 61–67. 10.3109/02841869509093640 (1995). 7865238 10.3109/02841869509093640

[30] Zhang, Q. et al. PUS7 promotes the proliferation of colorectal cancer cells by directly stabilizing SIRT1 to activate the Wnt/beta-catenin pathway. *Mol. Carcinog.* **62**, 160–173. 10.1002/mc.23473 (2023). 36222184 10.1002/mc.23473

[31] Du, J., Gong, A., Zhao, X. & Wang, G. Pseudouridylate synthase 7 promotes cell proliferation and invasion in colon cancer through activating PI3K/AKT/mTOR signaling pathway. *Dig. Dis. Sci.* **67**, 1260–1270. 10.1007/s10620-021-06936-0 (2022). 33811565 10.1007/s10620-021-06936-0

[32] Barchiesi, G. et al. Emerging role of PARP inhibitors in metastatic triple negative breast cancer. Current scenario and future perspectives. *Front. Oncol.* **11**, 769280. 10.3389/fonc.2021.769280 (2021). 34900718 10.3389/fonc.2021.769280 PMC8655309

